# HPLC-DAD Analysis and Investigation of Biological Properties of the Leaves of *Globularia alypum* (L.), Infusion Extract

**DOI:** 10.3390/ph16121726

**Published:** 2023-12-14

**Authors:** Sahar Nouir, Aicha Laouani, Ahmed Loghmari, Khaireddine Bouassida, Raoudha Slim, Rim Bouhajeb, Yosra Hasni, Khaled Saguem, Bouraoui Ouni, Amira Zaïri

**Affiliations:** 1 Laboratory of Biochemistry, Faculty of Medicine of Sousse, University of Sousse, Sousse 4023, Tunisia; sanouir@yahoo.fr; 2 Laboratory of Metabolic Biophysics and Applied Pharmacology (LR12/ES02), Faculty of Medicine of Sousse, University of Sousse, Sousse 4023, Tunisia; laouani_aicha@yahoo.fr (A.L.); khaledsaguem@famso.u-sousse.tn (K.S.); 3 USCR Analytical Platform UHPLC-MS & Research in Medicine and Biology, Faculty of Medicine of Sousse, University of Sousse, Sousse 4023, Tunisia; 4 Urology Department, Sahloul Hospital, Sousse 4054, Tunisia; loghmariahmed@gmail.com (A.L.); bouassidakhaireddine@gmail.com (K.B.); 5 Laboratory of Bioactive Natural Substances and Biotechnology Research, Faculty of Dental Medicine of Monastir, University of Monastir, Sousse 4023, Tunisia; raoudha_slim3@yahoo.fr (R.S.); elounibouraoui@yahoo.fr (B.O.); 6 Department of Pharmacology, Faculty of Medicine of Sousse, University of Sousse, Sousse 4023, Tunisia; rimbouhajeb1993@gmail.com; 7 Department of Endocrinology, Farhat Hached Hospital of Sousse, Sousse 4000, Tunisia; yosrahasnielabed@gmail.com

**Keywords:** *Globularia alypum* L., HPLC-DAD, antioxidant activity, anti-arthritic activity, antiproliferative effect, antibacterial and antibiofilm activities

## Abstract

*Globularia alypum* L. (GA) belonging to the Globulariaceae family is a Mediterranean plant which is widely used in traditional Tunisian medicine. The aim of this study was to investigate the phytochemical composition, antioxidant, anti-arthritic, antiproliferative, antibacterial and antibiofilm potential of aqueous GA leaf extracts (AGAL). Quantitative analyses of the different constituents of extracts were evaluated by high-performance liquid chromatography with photodiode-array detection (HPLC-DAD). Spectrophotometric methods and chemical tests were used for antioxidant and anti-arthritic activities. The antiproliferative study was evaluated using colorectal cancer SW620 cells, while the antibacterial assessment and analysis of the antibiofilm effects were determined by the microdilution method and the crystal violet assay, respectively. AGAL extracts presented several components, mainly Nepetin-7-Glucoside and trans-ferrulic acid. The results showed that they had an important antioxidant (IC_50_ = 0.34; 0.38 and 1.20 mg/mL) and anti-arthritic (IC_50_ = 2.94 mg/mL) properties, and these effects are displayed in a dose-dependent manner. In addition, this extract demonstrated significant antiproliferative (IC_50_ = 50 µg/mL), antibacterial (MIC = 6.25 mg/mL and MBC = 6.25 mg/mL), and antibiofilm (59.70% at 25 mg/mL) properties especially against *S. aureus*. The results achieved confirm the important role of this plant as a source of therapeutic activities.

## 1. Introduction

Medicinal plants can yield a wide range of bioactive substances, particularly secondary metabolites. Thus, numerous scientists have reported conducting in-depth investigations utilizing various plant extracts to evaluate the antibacterial, anti-inflammatory, analgesic, antioxidant, and many other therapeutic properties of these extracts [1]. A wild plant in the *Globulariaceae* family is *Globularia alypum* L. (GA). This shrub is perennial and can be found in the Mediterranean region. There are several uses for the plant [2]. It is one of the most popular traditional plant treatments in the Tunisian traditional pharmacopoeia and it is known by the local name Zrigua. Traditionally, its leaves have been employed as cholagogue, laxative, stomachic, purgative, and sudorific agents [3]. Additionally, ethnobotanical studies have demonstrated its effectiveness in the treatment of cardiovascular and renal illnesses [1].

Other studies showed that the active principles in leaves, stems and flowers in methanol and dichloromethane extracts of GA are supposed to antagonize the contractile response induced by different neurotransmitters [4]. More recently, GA petroleum ether extract showed anti-tuberculosis activity against *Mycobacterium tuberculosis* while a GA methanol extract has shown a potent antioxidant effect [5,6,7]. All these biological activities are mainly due to the abundance of secondary metabolites, such as polyphenols and iridoids [8]. It is also important to mention that several studies have stated the different activities of GA, but most of them deal with organic extracts, whereas only few studies report the bioactivities of their aqueous extracts, namely decoctions or infusions. In fact, it has been reported that the aqueous GA leaf extract (GAAE) has the potential for human male infertility treatment since the leaves contain active compounds with androgenic qualities that enhance active spermatogenesis in mice at a daily dose of 100 mg/kg for 15 days [9]. In addition, previous in vivo studies showed a laxative effect of GAAE in constipated rats, and an anti-inflammatory effect in rats with induced ulcerative colitis (UC) [10].

The hypoglycemic and hypotriglyceridemia roles are another potential of GAAE; this effect was shown in rats through repeated oral administration [4]. Additionally, it has been noted that the aqueous extracts are comparatively safe because a 10 g/kg dosage was not deadly in mice [9] and the GA LD50 value in rats was found to be above 14.5 g/kg [11]. GA aqueous extract has also been shown in other studies to have potential as an adjuvant for the management and/or prevention of oxidative stress and hypertriglyceridemia. Indeed, researchers demonstrated that offering rats fed a high-fructose diet an extra dose of GA aqueous extract protects hypertriglyceridemia while also lowering TG-VLDL levels. Furthermore, by raising SOD and GPx, GA can shield the heart from oxidative stress [12,13]. To the best of our knowledge, no data are available regarding antimicrobial or anti-arthritic activities of the aqueous extract of GA. Accordingly, the aim of the present study is to scrutinize the antioxidant, anti-arthritic, antiproliferative, and antimicrobial activities of aqueous extracts obtained by infusion of *G. alypum* leaves. Moreover, a phytochemical analysis of extracts was also carried out.

## 2. Results

### 2.1. HPLC-DAD Analysis

**Figure 1:**
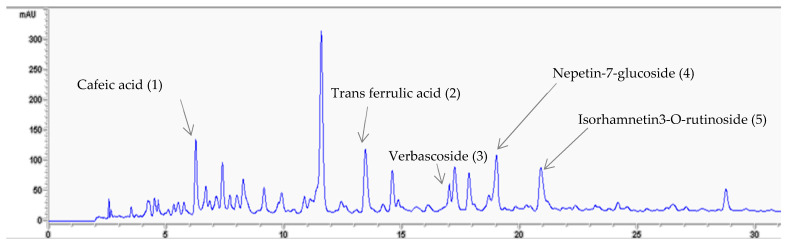
Chromatographic profile of *Globularia alypum* L. acquired at 324 nm.

**Figure 2:**
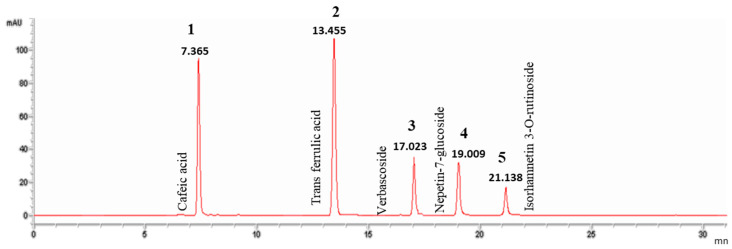
Chromatographic profile of standards recorded at 324 nm.

**Figure 3:**
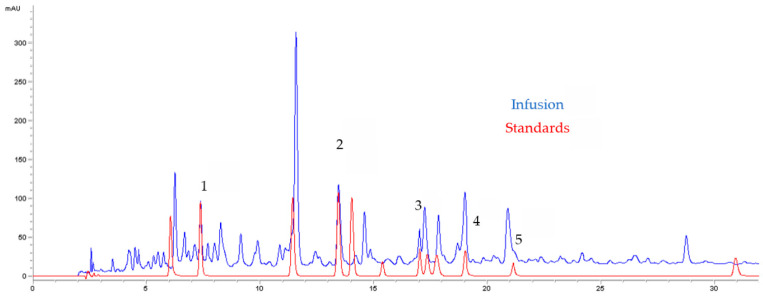
Chromatographic profile of *Globularia alypum* L. extract (blue) and standards (red) acquired at 324 nm.

An HPLC-DAD analysis system was used to separate and identify the active substances contained in GA infusion. The chromatographic separation of the infusion extract is shown in Figure 1. Analysis of this chromatogram recorded at 324 nm indicated the presence of phenolic compounds. Referring to the chromatogram of the standards (Figure 2), a total of five phenolic compounds were detected and identified on the basis of their retention times. The compounds were labelled according to their order of elution. The phenolic compounds detected in the GA infusion included Cafeic Acid: peak 1, RT = 7.4 min, Trans Ferrulic Acid: peak 2, RT = 13.4 min, Verbascoside:peak 3, RT = 17.04 min, Nepetin-7-Glucoside: peak 4, RT = 19.01 and Isorhamnetin-3-O-Rutinoside: pic 5, RT = 21.13 min (Table 1). Figure 3 illustrates the superposition of the two chromatograms, the infusion chromatogram and the chromatogram of the authentic standards purchased from Sigma Aldrich. In addition to retention time, peak identification is based on the agreement between the spectra of the analyte of interest and its corresponding authentic standard.

By recording the absorption spectra of the eluted analytes whose chromatographic peaks are illustrated in Figure 1, it is possible to distinguish three groups of analytes according to the shape and location of the spectral bands in their absorption spectra. The first group is formed by caffeic acid which represents an absorption spectrum characteristic of a phenolic acid chromophore. The second group is formed by Verbascoside and transferrulic acid; these compounds represent a characteristic absorption spectrum of a phenylpropanoid glycoside chromophore. The last group is formed by flavonoid glycosides represented by Nepetin7 glucoside and isorhamnetin 3-o rutinoside and whose absorption spectra are characteristic of a flavonoid chromophore.

In terms of quantity, nepetin-7-O-glucoside (30.82 μg/mL) was the most abundant component, followed by transferulic acid (10.33 μg/mL (*w*/*w*) (DW). Verbascoside and caffeic acid concentrations were present in smaller amounts in the plant infusion (7 μg/mL).

### 2.2. Antioxidant Activity

The antioxidant effect of *G. alypum*. infusion leaf extracts was performed by ABTS and β-carotene assays; the results were expressed as IC50 values. As presented in Table 2, the extract of *G. alypum* showed important antioxidant activities with significant variability between the used methods. The highest antioxidant potential with a value of IC50 = 0.24 ± 0.03 mg/mL was obtained by β-carotene assay, whereas a value of IC50 = 0.38 ± 0.02 mg/mL was obtained by ABTS. We concluded that the β-carotene activity assay was the most sensitive one in terms of IC50 by comparing the two methods used (Table 2).

### 2.3. Anti-Arthritic Effect

The results of in vitro anti-arthritic activity showed that aqueous extracts of *G. alypum* leaves had auspicious activity, as shown from the results on histamine releases, protein denaturation assay (Table 3).

### 2.4. Antiproliferative Effect

**Figure 4:**
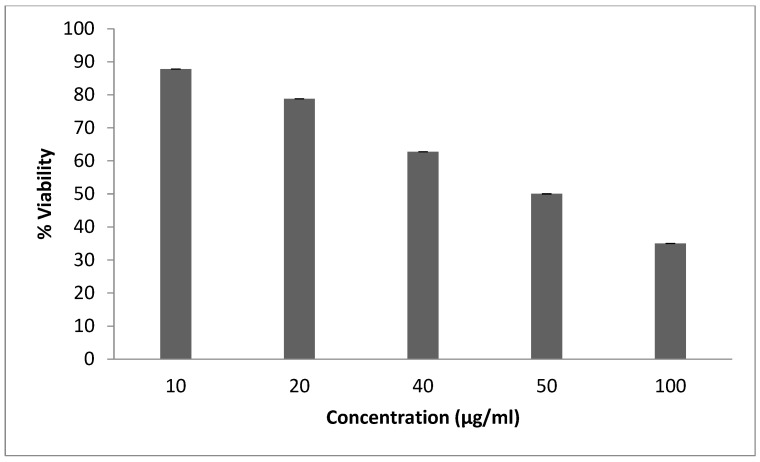
Antiproliferative effect of *G. alypum* leaves obtained by infusion. Viability rate (means ± standard deviations) of SW620 cells treated with the extract obtained by infusion of *G. alypum* leaves. (IC_50_ = 50 µg/mL); IC_50_: Concentration required reducing SW620 cell viability by 50%.

In the present study, an evaluation of the antiproliferative effect of different extracts from *G*. *alypum* leaves on SW620 was performed at various concentrations. After 48 h of incubation, the survival rates showed that all the extracts inhibit cell line proliferation in a concentration-dependent manner. The infusion extract presented a higher anti-proliferative effect with an IC50 = 50 µg/mL (Figure 4).

### 2.5. Evaluation of Antibacterial Activity

Table 4 and Table 5 demonstrate the aqueous *G. alypum* extracts’ capacity to suppress the growth of both Gram-positive and Gram-negative planktonic cells. All the tested stains exhibited sensitivity to the extract, according to the results. *S. aureus* had the strongest effect, with a minimum inhibitory concentration (MIC) of 6.25 mg/mL and a zone of inhibition measuring 24 mm.

### 2.6. Antibiofilm Activity

In the current study, the antibiofilm effect of infusion extracts of *G. alypum* were tested at different concentrations (MIC and 1/8 MIC). The biofilm inhibition rates against *S. aureus* were 45.39% to 59.70%, and 31.69 to 45.99% against *S. thyphimirium*. Moreover, the biofilm inhibition was weaker for *E. coli* and totally absent for *P. aeuroginosa*. It is worth noting that *G. alypum*, which strongly affected *S. aureus* and *S. thyphimirium* biofilm, was the least active against *P. aeuroginosa* (Table 6).

## 3. Discussion

Many substances found in nature, referred to as secondary metabolites, are derived from plants and have been used for millennia to create new therapeutics. In general, the phytochemical investigation of *Globularia alypum* revealed the presence of significant amounts of various chemical components, while the present work is aimed at analyzing the aqueous extracts of leaves of *G alypum* using the HPLC-DAD method for detecting the characteristic peak values and their functional groups. HPLC analysis revealed different characteristic peak values with various functional constituents in the extracts. The identified chemicals in Tunisian GA extracts have never been reported. The examined extracts were different from those in the results of other researchers working on Tunisian aqueous *G. alypum* species. Indeed, the phenolic fraction of *Globularia alypum* aqueous extract (GAAE) from the northwest of Tunisia is dominated by iridoids and secoiridoids with an abundance of serratoside, as demonstrated by Hajji et al. utilizing HPLC-PDA/ESI-MS analysis. They demonstrated the detection of three more flavonoids. Phellamurin comes in third, after quercetin glucoside and gallo-catechin as the primary ingredients. Verbascoside constituted the majority of GAAE’s composition [10]. Furthermore, since the aqueous extracts of Bouriche et al. include distinct components, their study can be cited. In fact, the authors noted that the aqueous extracts of *G. alypum* that were collected in June 2010 from Sétif, in Eastern Algeria, showed the presence of flavonoids and phenolic acids according to the HPLC-TOF/MS analysis. The glycoside flavonoids diosmin, rutin, and scutellarin are present in significant concentrations in these extracts, with naringin and quercetin-3-β-D-glucoside having the highest concentrations. Additionally, they disclosed that of the identified phenolic acids, there was a high cinnamic acid concentration together with protocatechuic acid [14]. The variations observed in the phenolic profiles of aqueous extracts derived from diverse sources may be attributed to growth and environmental factors such as soil composition, altitude, rainfall, and climate, as well as the distinct methods employed to ascertain this composition. These elements may have a direct impact on the composition of chemical components. Given their high phenolic component content, our obtained extracts will probably show a range of biological activity [13]. For instance, Calvin et al. reveled that nepetin isolated from *Eupatorium arnottianum* (EA) was investigated in the TPA mouse ear edema and was found to be active. Nepetin reduced the TPA mouse ear edema and inhibited the NF kappaB induction as it is a major component of the EA extract [15]. Moreover, Boubaker et al. showed that isorhamnetin 3-O-rutinoside promotes apoptosis of human myelogenous erythroleukaemia cells [16]. Furthermore, as far as we know, few studies have been conducted to determine the phenolic composition of aqueous extracts of *Globularia alypum* in Tunisia. The evaluation of these extracts is very limited; only few plants have been evaluated to date, and no data have been described before to determine the polyphenol composition of decoction or infusion extracts. Almost all the available studies in the literature reported mostly on methanolic extracts or essential oils.

Nowadays, due to safety concerns for synthetic antioxidants, there is an increasing demand for natural antioxidants [16]. Herbs are great sources of antioxidants due to their natural antioxidant components, especially for food preservation. The results shown in this study revealed that aqueous extracts of *G. alypum* leaves showed important antioxidant activities and that this effect was in a dose-dependent relationship. Regarding antioxidant activity, it has been previously reported that phenolic compounds such as flavonoids, phenolic acid, and tannins contribute considerably to the antioxidant activity in medicinal plants [17]. Our extract contained the highest-level nepetin-7-O-glucoside. It has been reported that this molecule exhibits several pharmacological effects, including anti-inflammatory and antioxidant activities [15]. In addition, isorhamnetin 3-O-rutinoside is a flavonoid glycoside that exerts an antioxidant effect [16]. Our results are also in compliance with a study conducted by Jrah et al., who showed that aqueous extract of *G. alypum* showed the highest antioxidant capacity with a value of 8.9 mM TE and 4.5 mM TE by FRAP and the Reducing Power (RP) method, respectively [17]. It is interesting to note that our findings imply that our extracts have noticeably more antioxidant activity than other nearby plants. For instance, according to ABTS and β-carotene assays, *G. alypum* obtained from Oueslatia Kairouan in central Tunisia showed antioxidant activity at IC 50 = 0.89 and 0.42 mg/mL, respectively [18]. On the other hand, 0.04 and 0.15 mg/mL were sufficient to enhance the same impact. Additionally, a study conducted in January 2016 in Batna, Algeria examined the antioxidant activity of *G. alypum* collected from Ain Zaatot. Two methods were used to quantify this activity: the ferric reducing antioxidant power (FRAP) and the DPPH radical scavenging assay. The data obtained indicated that *G. alypum* exhibited significant concentrations of scavenging activity: IC50 223.04 ± 0.73 to 85.37 ± 1.48 µg/mL and EC50 0.31 ± 0.01 to 0.68 ± 0.19 mg/mL [19]. The variety seen in the antioxidant activities of G. alypum might likely be explained by the experimental setup, the methods employed, and the utilization of plants from various geographical regions. It is important to mention that aqueous extracts of *G. alypum* showed an effect on colonic antioxidant enzyme activity. A study conducted by Hajji et al. showed that GA collected from Northwest of Tunisia had a significant decrease in superoxide dismutase (SOD) and gluthation peroxidase (GPx) activities in the colon of AA-intoxicated animals compared to the controls, whereas GAAE (100, 200 and 400 mg/Kg, b.w.) or sulfasalazine (100 mg/Kg, b.w.) pretreatment over seven days significantly protected against this depletion when compared to the colitis group (*p* < 0.05) [10]. In the same context, a recent study of Hajji et al. proved that GAAE regenerates antioxidant enzymes after depletion under LOP administration enzyme levels showed depletion when treated only with LOP. However, administration of yohimbine regenerates antioxidant enzyme levels. GAAE also significantly increased SOD and GPx levels in a dose-dependent manner (400 mg/kg) [13]. The results of a second Algerian investigation demonstrated that *G. alypum* methanolic and aqueous extracts, which were obtained in June 2010 from Sétif in Eastern Algeria, could chelate ferrous ions in a concentration-dependent way. In contrast to the methanolic extract, the aqueous extract displayed more activity. In total, 52.69 μg/mL and 148.15 μg/mL, respectively, were the IC50 values. Compared to the results obtained with EDTA, the standard chelator, which produced an IC50 value of 5.97 μg/mL, this activity was less significant [14]. We draw the conclusion that the primary cause of the phenolic components of GA’s antioxidant activity is their redox characteristics, which enable them to function as singlet oxygen quenchers, hydrogen donors, and reducing agents [20]. A metallic chelating potential might also exist in them [21].

According to WHO, 0.3–1% of the world population is affected by rheumatoid arthritis (RA), and among them females are three times more prone to the disease compared to males [22]. RA is a chronic, inflammatory, and systemic autoimmune disease [23]. Therapeutic agents against arthritis such as analgesics and NSAIDS reduce inflammation and joint destruction in either acute or chronic RA patients [24]. On the other hand, gastrointestinal ulcers, cardiovascular complications, myelosuppression, hepatic fibrosis, stomatitis, cirrhosis, nephrotoxicity, pulmonary toxicity, immunological reactions, and local injection-site reactions are among the long-term dangers associated with medication use. Furthermore, increased expenses and adverse effects, such as elevated chances of infections and miscarriages, necessitate ongoing surveillance [24]. As a result, fresh strategies are created to preserve the harmony between these possible hazards and recognized advantages [25]. Safer and more effective medications are currently being created from oriental sources for the treatment of RA. To lessen these negative effects and boost the positive effects, a wide range of herbal extracts and products, including polyherbal formulations, are created [24]. A review conducted by Choudhary et al. reported that about 485 plant species would have a promising anti-arthritic activity in humans. Cross-validation of information regarding the ethnic proof of historically used anti-arthritic plants was conducted using a variety of journal articles and reviews [26]. As far as we know, no such review has examined the relationship between the plant family, components utilized, and dosage form and the anti-arthritic properties of the plants. According to these data, the Papilonaceae family of plants has a higher concentration of plants with anti-arthritic action. Of these sections, leaves have been utilized most frequently in oil dose form to treat arthritis [26]. The used extracts in this study showed that aqueous extracts of *G. alypum* had an important anti-arthritic activity. In light of our knowledge, the evaluation of anti-arthritic activity of *G. alypum* is extremely limited. Only a little data relating to this activity have been reported. For instance, Friscic et al. showed that 50 μg/mL of aqueous extracts of *G. alypum* inhibited COX-1 activity and that this inhibition reached 51.3% (*G. alypum*) in the TMPD assay and 40.6% in the PGE2 assay. The authors also showed that the results obtained for *G. alypum* are comparable to those reported for the methanolic leaf (GAME) (5.33%) and the flower extract (61.05%) of the same species tested at a 33 μg/mL concentration using the TMPD assay [27]. Another study reported that the treatment of animals with GAME produced a significant decrease in the inflammatory cells; however, slight improvements in edema tissue were observed in the reference group. The authors explained that this anti-inflammatory effect of GAME may be equally related to its cinnamic acid content [28]. Additionally, they demonstrated that the outcomes for *G. alypum* are similar to those reported for the same species’ methanolic leaf (GAME) (5.33%) and flower extract (61.05%) when examined using the TMPD assay at a dose of 33 μg/mL [27]. Another study found that offering GAME to rats resulted in a considerable reduction in inflammatory cells, although the reference group’s edema tissue showed only modest reductions. The authors clarified that GAME’s anti-inflammatory properties might be equally attributed to the amount of cinnamic acids it contains [28].

To select plant extracts with potential antitumor properties for future bio-related studies, cytotoxicity screening models provide important preliminary data [17]. To date, no reports have been found in the literature on the evaluation of the cytotoxicity of aqueous leaves extracts, as performed in this study. The present study examined, for the first time, the ability of AGAL to inhibit cancer cell proliferation, which is known to be the most promising route to treat cancer. The infusion extract presented a higher anti-proliferative effect with an IC50 = 50 µg/mL. This result can be explained by the richness of this extract of active molecules, especially ferulic acid (Figure 3). In fact, the study of Ekowati et al. performed in 2020 demonstrated that ferulic acid showed anticancer potential by suppressing angiogenesis and causing inhibition of melanoma growth in a xenograft model [29]. Comparing our result with the study of Friscic et al. [27], we distinguished that our extract has the lowest antiproliferative effect (IC_50_ = 50 and 231.43 µg/mL). In addition, no research has been performed previously to evaluate the anti-proliferative activity of aqueous extract.

Nowadays, many researchers around the world are working on plant extracts to develop new antimicrobial agents with enhanced safety and efficiency [15,16,17,18,19]. The prime objective of ethnopharmacology is to identify plants for medicinal importance with minimal side effects. Additionally, active compounds from the plant extracts with antibacterial activity can be transformed into possible medication. Research to develop efficient and accessible medication from active plant compounds in the interest of public health is the need of the present world. The present in vitro experimental study explored the antibacterial effect of aqueous extracts of *G. alypum* leaves against *Pseudomonas aeruginosa*, *Salmonella enterica* subsp. *enterica serovar Typhimurium*, *Escherichia coli*, and *Staphylococcus aureus.* The tested stains exhibited sensitivity to the extract, according to the results. *S. aureus* had the strongest effect, with a minimum inhibitory concentration (MIC) of 6.25 mg/mL and a zone of inhibition measuring 24 mm. The discussion is limited because, although the anti-arthritic effect of *G. alypum* aqueous extracts has been previously described, no research has been conducted to characterize its antibacterial activity. The limited information that is currently available on this activity, however, mostly reports on the antibacterial action of organic extracts [30]. For instance, we previously reported that sonication-obtained extracts of *G. alypum* demonstrated a noteworthy antibacterial effect on *S. aureus*, exhibiting a zone of inhibition of 14.5 mm that aligns with our actual study. However, these methanolic extracts demonstrated no effect against the remaining strains tested [31]. In addition to other water-soluble components that were naturally present in the plant material, Nayak et al. speculated that the anionic components, such as chlorides, thiocyanate, sulfates, and nitrate, may be responsible for the antibacterial action of aqueous extracts [32].

Since polyphenols, tannins, and flavonoids are often produced by plants and are involved in their defense against microbial infections, their presence in plant extracts may account for the antibacterial activity. Interestingly, Nepetin-7-Glucoside, a flavonoid and glycoside, is highly abundant in our extract [33]. Furthermore, trans-ferulic acid (TFA), which is abundant in the aqueous extract, possesses a variety of pharmacological qualities, such as antifungal and antibacterial activities [34].

In compliance with several other studies, the results obtained in this study showed that Gram-positive bacteria are more susceptible to plant extracts than Gram-negative bacteria. These differences may be related to the cell wall composition of bacteria. In fact, the cell wall of Gram-positive bacteria is single layered, whereas that of Gram-negative cells is multilayered [35]. Moreover, our extract also inhibited Gram-negative bacteria. This can be explained by the fact that Gram-negative bacteria have a hydrophilic membrane due to the presence of lipopolysaccharides, and thus, a small hydrophilic molecule can pass through the outer membrane as it was reported in several studies [35]. On the contrary, lipophilic compounds and macromolecules can pass through this outer membrane. Therefore, understanding of the permeation properties of the outer membrane of the microorganisms is essential to know about the antibacterial activity of a solute [35].

Extracellular polysaccharides (EPS) are a complex matrix of microorganisms that link to one another to form biofilms. These biofilms can shield cells from antibacterial agents and decrease their antibacterial effectiveness [36]. Since adhesion affects the biofilm’s eventual development and maturity, it is the initial and most important stage in the formation of a biofilm [37]. Naturally occurring bacteria that produce biofilms are the main source of many illnesses in both humans and animals [38]. Bacterial biofilms can also result in major issues for the food business. These days, a lot of research has been conducted with the aim to create benign antibiofilm agents because they do not eventually induce medication resistance [39]. In the current study, an antimicrobial assay showed that our extracts displayed an antibiofilm activity especially against *S. aureus*. To the best of our knowledge, no prior information has been provided regarding the antibiofilm activity of *G. alypum*, and this is the first time that it has been documented. Furthermore, our investigation demonstrated that *G. alypum* extract exhibited clear antibiofilm efficacy against *S. aureus* biofilms at doses equal to or lower than the 1/4 MIC threshold. Many phytochemicals found in medicinal plants, including flavonoids, glycosides, tannic acid, phenolics, and chlorogenic acid, have the ability to suppress or completely eliminate biofilms by preventing the growth of bacteria that produce them, rupturing the polysaccharides in extracellular polymer (EPS), destroying the integrity of the bacteria’s membrane, preventing the activity of enzymes linked to biofilm formation, rupturing the fibrils that allow bacteria adherence to one another, suppressing the expression of genes related to biofilms, or suppressing the quorum-sensing system [40,41,42].

*G. alypum* contain more than a dozen chemical constituents, including the following flavonoids: cafeic acid, trans-ferrulic acid, Verbascoside, Nepetin-7-Glucoside, Isorhamnetin-3-O-Rutinoside and other chemical constituents. We hypothesize that the combined action of *G. alypum*’s phytoconstituents, particularly the abundant flavonoids and ferrulic acid that have demonstrated both biofilm and antibacterial activity, may be responsible for both the suppression of biofilm formation and antibacterial activity. Nevertheless, a thorough explanation of the precise mechanisms at play is still required. We already demonstrated the high ferulic acid content of our extracts. One of the most prevalent phenolic acids in natural species is ferulic acid (AF), also known as 3-(4-hy-droxy-3-methoxyphenyl)-2-propenoic acid, a secondary metabolite that is a member of the phenolic chemical class [43]. After indirect studies demonstrated that ferulic acid had a high antibacterial activity against *L. monocytogenes* [44], the examination of ferulic acid’s antimicrobial potential was proposed. The study of Pinheiro and his collaborators demonstrated that ferulic acid derivatives inhibit *Staphylococcus aureus* [45].

## 4. Materials and Methods

### 4.1. Plant Material

The study’s use of plant material adheres to appropriate institutional, national, and international rules and regulations. *G*. *alypum* was collected at the full blooming stage in Tunisia’s Ouardanin area in January 2019 (Table 7). Prof. Fethia Harzallah Skhiri of Tunisia’s High Institute of Biotechnology in Monastir completed taxonomic identification. The plant voucher specimen was cataloged as Ga 022 in the Herbarium of the Laboratory of Bioresources: Integrative Biology and Valorization (ISBM). For 7 days, the leaves were washed and dried at room temperature. The material was dried until it reached a constant weight. To optimize phenolic extraction, the leaves were milled.

### 4.2. Preparation of the Infusion

A total of 5 g of shade-dried plant was soaked for 30 min in 100 mL of boiling distilled water. The infusion was then filtered through Whatman No. 4 paper, lyophilized and stored in an amber glass container at 4 °C until use.

### 4.3. HPLC-DAD Analyses

The chromatographic method adopted for analyses is based on a detection set at 324 nm. The Agilent 1100 Series HPLC system supplied with a diode array detector (DAD) is used. The chromatographic device is a computer-controlled agilent 1200. A reversed-phase Kinetex Evo C18 column placed in an oven at 30 °C is used for the separation of the different analytes in the infusion extract. The mobile phase consists of a mixture of water, acetonitrile and formic acid. Mobile phase A is formed by a combination of acetonitrile and formic acid (1%). Mobile phase B is formed by 99% water and 1% formic acid. The gradient program is conducted in the following way: 10% A, 90% B (0 min), 20% A, 80% B, (20 min), 25% A, 75% B (30 min), 35% A, 65% B, (40 min) and 10% A, 90% B10 (50 min). The rate of mobile phase elution is 1 mL/minute. Samples are filtered using a 0.20 μm filter prior to injection. The injected volume is 20 μL. Identification of the peaks of phenolic compounds is obtained by comparing their retention times as well as their UV spectra with those of available authentic standards [46,47]. All standards are purchased from Sigma-Aldrich. Peak identification is performed by congruent retention times and UV spectra compared to available authentic standards. Quantification is estimated by comparing the area of the peak of interest with that obtained in a standard chromatogram corresponding to a known concentration.

### 4.4. Antioxidant Activity

The method of El Arem and his collaborators based on spectrophotometric analysis was used to trap ABTS^+^ cations [47]. To generate the ABTS radical cation, equal volumes of a solution of potassium persulfate K_2_S_2_O_8_ 16 h prior to use and a stock solution of ABTS (39.2 mg) were mixed, kept at room temperature, and stored away from light. After that, ethanol was added to the solution to dilute it until the absorbance at 734 nm was between 0.7 and 0.8. For each analysis series, a volume of 975 µL of this freshly made solution was added to 25 µL of each extract, and the reading was taken at 734 nm after 20 min. A positive control was ascorbic acid. Based on the solution’s discoloration, the antioxidant activity was calculated and expressed as the percentage inhibition (PI) of absorbance at 734 nm, the wavelength at which the ABTS+• radical exhibits a distinctive absorption band. Inhibition (%) = [(Abs control − Abs extract)/Abs control] × 100. All assays were performed in triplicate.

### 4.5. β-Carotene Bleaching Assays

The production of peroxide radicals from the oxidation of linoleic acid led to the oxidation of β-carotene, resulting in the loss of its red hue. An emulsion containing 0.5 mg β-carotene, 25 µL linoleic acid, 1 mL chloroform, 200 mg Tween 20-, and 100 mL distilled water served as the basis for this test. Subsequently, 2.5 mL of this emulsion was mixed with 0.5 mL of the extract or a control BHT (butyl hydroxytoluene) and incubated in a water bath at 50 °C for two hours. After adding the emulsion, the sample was tested many times at 20 min intervals [48], with the absorbance at T0 being recorded at 490 nm. Using the following formula, the percentage of inhibition of peroxidation was determined: IP = β-carotene after 2 h/initial β-carotene × 100. All assays were performed in triplicate.

### 4.6. Anti-Arthritic Effect

Using bovine serum albumin (BSA), the anti-arthritic effect of AGAL on the prevention of protein denaturation was assessed. The reaction mixture (0.5 mL) included 0.05 mL of various AGAL concentrations (0.0039–10 mg/mL) and 0.45 mL of BSA (5% aqueous solution) in addition to acetyl salicylic acid (reference drug), in that order. Using 1 N HCl, each solution was adjusted to pH 6.3. After adding 2.5 mL of phosphate buffer and incubating the samples for 30 min at 37 °C, the absorbance of the samples was determined using a spectrophotometer at 660 nm. *G. alypum* was replaced with 0.05 mL of distilled water for the test control, and BSA was absent from the product control [49]. The percentage inhibition of protein denaturation was deliberated by the following formula: Percentage inhibition = (Abs_Control_ − Abs extract)/Abs_Control_) × 100.

### 4.7. Antiproliferative Effect

Using a measurement of the SW620 cells extracted from colorectal human cells, the antiproliferative effect was assessed using the MTT assay for cellular metabolic activity. These cells were fixed at a density of 5 × 10^3^ on a plate (MultiScreen^®^filtration plates, 96-well plates) in an incubator set at 37 °C with 5% CO_2_. After the cells were taken out of the medium culture, they were treated with the extracts at various concentrations (100, 50, 40, 20, and 10 µg/mL) that were diluted in DMSO. This experiment was conducted three times. After 48 h of incubation, the MTT solution (1 mg/mL) treatment was started in RPMI 1640 culture media supplemented with 10% fetal bovine serum and a 10 µg/mL antibiotic: 5 µg/mL penicillin and 5 µg/mL streptomycin. The formation of purple formazan crystals from the yellow tetrazolium salt served as a marker for cells that were engaged in metabolism. After two hours of incubation at 37 °C with 5% CO_2_, we evaluated the ratio (OD in the test group/OD in the control group × 100) using an ELISA reader to determine the percentage of survival of these cells. Three separate experiments were carried out.

### 4.8. Antibacterial Activity

The following types of bacteria were tested for AGAL’s antibacterial activity using the agar disc diffusion method [50]: *Pseudomonas aeruginosa* ATCC 27853, *Salmonella enterica* subsp. *enterica serovar Typhimurium*. ATCC 14080, *Escherichia coli* ATCC 25922, and *Staphylococcus aureus* ATCC 25923. Using a sterile cotton mop, the pathogenic bacteria inoculums were adjusted to a 0.5 McFarland standard turbidity before being streaked onto Muller–Hinton (MH) agar plates. The agar mediums were covered with sterile filter discs (diameter 6 mm, Biolife, Rome, Italy), onto which 20 µL of each extract, diluted at a concentration of 25 mg/mL in sterile distilled water, was dropped. As a reference antibiotic, tetracycline (10 mg/mL; 10 µL/disc) was employed. The inhibition zone was measured to assess the antibacterial activity following a 24 h incubation period at 37 °C. Every assay was run three times. A MIC assessment was conducted [51]. The target bacteria and MH broth were added to plates containing 96 U-bottomed wells (Nunc, Roskilde, Denmark) together with serial dilutions of the extracts (0.05–25 mg/mL). Following a 24 h incubation period at 37 °C, the MBC was assessed by transferring 10 µL of the well’s contents, which revealed no bacterial growth following the MIC test on MH agar. Upon examining the bacterial growth over a 24 h incubation period at 37 °C, the MBC was identified as the sample concentration with the lowest bactericidal action. Every assay was run in triplicate.

### 4.9. Antibiofilm Assay

The antibiofilm assay was determined using the crystal violet assay [51]. Various extract dilutions were produced at varying concentrations (0.05–25 mg/mL) in sterile distilled water. Subsequently, the mixture was added to plates containing 96 U-bottomed wells (Nunc, Roskilde, Denmark) that contained brain heart infusion (BHI) (Oxoid) with 2% glucose (*w*/*v*), containing pathogenic bacteria suspension (grown in BHI for 24 h at 37 °C; 105 CFU/mL). The negative and positive controls were BHI with 2% glucose alone and BHI with 2% glucose infected with the pathogenic strain, respectively. Following a 24 h incubation period at 37 °C, the plates underwent three PBS washes. The biofilm’s cells were air-dried, fixed with methanol for 15 min, then stained with 1% crystal violet. Using a microplate reader (GIO. DE VITA E C, Roma, Italy), the absorbance at 595 nm was used to quantify the production of biofilms. Three separate experiments were conducted, and the formula used to calculate the percentage of inhibition was as follows: (OD_control_ − OD_Extract_)/OD_control_ × 100 = Inhibition (%).

### 4.10. Statistical Analysis

The findings are shown as mean values ± standard deviation. Version 22 of SPSS was the foundation for statistical analyses. These analyses made use of the Student–Newman–Keuls test and ANOVA. For statistical significance, data were deemed different when the *p*-value was 0.05 or below.

## 5. Conclusions

The current investigation sheds more light on *G. alypum*’s phytochemical potential. The bioactive compounds found in the infusion extracts under investigation and their observed biological properties align with the findings of prior studies on biological activity. Additionally, these compounds offer significant health benefits not only in the treatment of oxidative stress-related diseases and reactive species production, but also in the prevention of bacterial infections.

## Figures and Tables

**Table 1:** Identification and quantification of five phenolic compounds of *Globularia alypum* L. extract acquired at 324 nm.

Peak	Retention Time (min)	Identification	Area	Quantity (µg/mL)
1	7.4	Cafeic Acid	468.3	7
2	13.4	Trans Ferrulic Acid	1011	10.33
3	17.04	Verbascoside	214.24	7
4	19.01	Nepetin-7-Glucoside	885.8	30.82
5	21.13	Isorhamnetin-3-O-Rutinoside	151.8	8.9

**Table 2:** Antioxidant activity of *G. alypum* leaves. Values with different superscripts within the same column are significantly different (*p* < 0.05), and each data point is represented by the average of three repetitions ± SD of one independent experiment; IC_50_: Half maximal Inhibitory Concentration.

IC_50_ (mg/mL)	Ascorbic Acid	Infusion
ABTS scavenging assay	0.06 ± 0.02 ^a^	0.38 ± 0.02 ^b^
β-carotene bleaching assay	0.59 ± 0.00 ^a^	0.24 ± 0.03 ^b^

**Table 3:** Evaluation of Anti-arthritic activity of *G. alypum* leaves. Results of the ANOVA test are significantly different at *p* < 0.05, and each data point is represented by the average of three repetitions ± SD of one independent experiment; IC_50_: Half maximal Inhibitory Concentration. Values with different superscripts within the same column are significantly different (*p* < 0.05).

IC_50_ (mg/mL)	Acetyl Salicylic Acid	Infusion
Protein denaturation	0.73 ± 0.02 ^a^	3.58 ± 0.02 ^b^

**Table 4:** Evaluation of antibacterial activity of *G. alypum* leaves at 25 mg/mL. Values are means of triplicate determination, (n = 3) ± standard deviation. N, no zone of inhibition was found.

Zone of Inhibition (mm)		
Strains	Tetracycline	Infusion
*E. coli*	29.10 ± 0.00	21.00 ± 0.00
*P. aeruginosa*	27.20 ± 0.53	11.50 ± 1.01
*S. typhimurium*	25.01 ± 1.02	21.50 ± 0.31
*S. aureus*	21.00 ± 1.82	24.00 ± 1.31

**Table 5:** MIC and MBC determination of the infusion extract of *G. alypum* leaves. Minimum Inhibitory Concentration (MIC) and Minimum Bactericidal Concentration (MBC). N: not found.

Strains	Tetracycline		Infusion	
Concentrations (mg/mL)	MIC	MBC	MIC	MBC
*E. coli*	3.12	6.25	>25	N
*P. aeruginosa*	3.12	6.25	>25	N
*S. typhimurium*	6.25	6.25	>25	N
*S. aureus*	6.25	6.25	6.25	6.25

**Table 6:** Percentage of biofilm inhibition after 24 h. Data are the means ± SD of three independent experiments. The values with different superscript letters show significantly (*p* < 0.05) different means.

Percentage of Inhibition (%)				
Concentrations (mg/mL)	*E. coli* ^a^	*S. typhimurium* ^b^	*S. aureus* ^c^	*P. aeruginosa*
25	26.31	45.85	59.83	N
12.5	18.94	38.68	54.92	N
6.25	16.19	37.21	53.11	N
3.12	14.23	31.15	45.31	N

**Table 7:** Collection site of *Globularia. alypum* L. and its eco-geographical characteristics.

Collection Site	Geographical Location		
	Longitude (E)	Latitude (N)	Altitude (m)
Ouardanin	10°40′35″	35°42′35″	75

## Data Availability

Materials, data and associated protocols are promptly available to readers without undue qualifications in material transfer agreements. Concerning data information, please contact corresponding author.
